# Modeling brain, symptom, and behavior in the winds of change

**DOI:** 10.1038/s41386-020-00805-6

**Published:** 2020-08-28

**Authors:** David M. Lydon-Staley, Eli J. Cornblath, Ann Sizemore Blevins, Danielle S. Bassett

**Affiliations:** 1 Department of Bioengineering, University of Pennsylvania, Philadelphia, PA 19104 USA; 2 Annenberg School for Communication, University of Pennsylvania, Philadelphia, PA 19104 USA; 3 Neuroscience Graduate Group, University of Pennsylvania, Philadelphia, PA 19104 USA; 4 Department of Psychiatry, Perelman School of Medicine, University of Pennsylvania, Philadelphia, PA 19104 USA; 5 Department of Neurology, Perelman School of Medicine, University of Pennsylvania, Philadelphia, PA 19104 USA; 6 Department of Electrical & Systems Engineering, School of Engineering & Applied Science, University of Pennsylvania, Philadelphia, PA 19104 USA; 7 Department of Physics & Astronomy, College of Arts & Sciences, University of Pennsylvania, Philadelphia, PA 19104 USA; 8 The Santa Fe Institute, Santa Fe, NM 87501 USA

**Keywords:** Cognitive neuroscience, Medical research

## Abstract

Neuropsychopharmacology addresses pressing questions in the study of three intertwined complex systems: the brain, human behavior, and symptoms of illness. The field seeks to understand the perturbations that impinge upon those systems, either driving greater health or illness. In the pursuit of this aim, investigators often perform analyses that make certain assumptions about the nature of the systems that are being perturbed. Those assumptions can be encoded in powerful computational models that serve to bridge the wide gulf between a descriptive analysis and a formal theory of a system’s response. Here we review a set of three such models along a continuum of complexity, moving from a local treatment to a network treatment: one commonly applied form of the general linear model, impulse response models, and network control models. For each, we describe the model’s basic form, review its use in the field, and provide a frank assessment of its relative strengths and weaknesses. The discussion naturally motivates future efforts to interlink data analysis, computational modeling, and formal theory. Our goal is to inspire practitioners to consider the assumptions implicit in their analytical approach, align those assumptions to the complexity of the systems under study, and take advantage of exciting recent advances in modeling the relations between perturbations and system function.

## Introduction

In the Dancing Forest on the Curonian Spit in Kaliningrad Oblast, Russia, dozens of pine trees loop around in rings, spirals, hearts, and squiggles. Some inquisitive minds have suggested the work of shifting sands or of *Rhyacionia buoliana* caterpillars, whereas a naive observer might instead turn to the whims of a capricious wind to explain the dances seemingly frozen in time. And precisely how might wind perturb a tree? The answer depends upon the nature of the form “tree”. If a tree were simply a trunk, resting on the ground, then a gust of wind could quite easily raze the tree. If, instead, a tree were a trunk with roots grasping the dirt, then a gust of wind might require additional energy to raze the tree, disturbing the dirt in the process. Finally, if a tree were not standing alone but in a forest of other trees characterized by an intertwined root system, then a gust of wind might require even more energy to raze the tree, disturbing both the dirt and the surrounding trees.

The thought experiment of tree and wind may initially seem esoteric. But upon closer inspection, open questions about the Dancing Forest display marked similarities to questions of fundamental import to neuropsychopharmacology. The tree we care about is a human brain, or a human symptom of psychopathology, or a human behavior. A wind may perturb the tree, either to raze it or twist it, into something beautiful or something broken. In common parlance, that wind may be a change in environment or context, a life event, a healthy or altered neurodevelopmental process, a pharmacological treatment, or a stimulation regimen. Our capacity to predict the beneficial or detrimental impacts of perturbations on brain, symptom, or behavior, depends in part upon the depth of our understanding of the nature of the form “brain”, or “symptom”, or “behavior”.

And what is the nature of the form “brain”? or “symptom”? or “behavior”? In some studies, we throw up our hands, admitting that we still cannot answer. We instead attempt simply to measure that nature. In other studies, we take a pragmatic tack by assuming a particular nature, thereby allowing us to subsequently make particular inferences. As the tree is assumed to be solely a trunk, a brain region is assumed to be an isolated volume with no connection to other brain regions; a symptom is assumed to be a self-reported experience with no dependence upon other experiences; and a behavior is assumed to be a response to a task with no relation to other responses. Such assumptions implicit in our experimental setup and analysis directly influence the type of inferences that we can draw from our results: the strength of the wind that we predict to be necessary to dance the tree.

Here we consider three common assumptions implicit in our analysis of empirical data in the spheres of brain, symptom, and behavior. We clarify how those assumptions correspond to existing formal models, each of increasing complexity: a commonly applied form of the general linear model (GLM) (tree trunk), impulse response models (tree trunk with roots), and network control models (forest). In each section, we first describe the model with brevity and simplicity. Next, we review how the model has been used in recent literature to understand neural, symptom, and behavior systems. Finally, we address the strengths and weakness of each model. Our presentation motivates a discussion of future directions in both basic and clinical science, and a thoughtful examination of the relative merit of different points along the continuum from machine learning to computational modeling to theory. The nature of our contribution is partly organizational; we offer the reader an organized account of existing and emerging computational models generically relevant to open challenges in neuropsychopharmacology. Moreover, we provide readers with conceptual tools to reason about their underlying assumptions.

## The systems relevant for neuropsychopharmacology

**Fig. 1:**
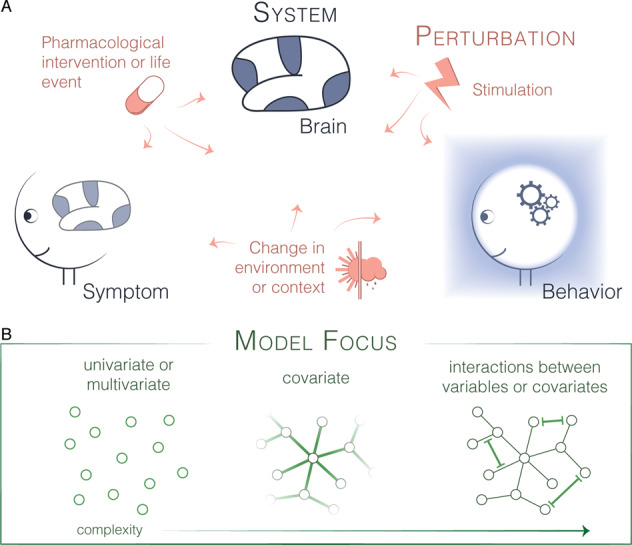
Overview of systems, perturbations, and models. **a** Here we depict the three systems (blue) of brain, symptom, and behavior. Perturbations (rose) including stimulation, pharmacological interventions, life events, and changes in environment can affect each. **b** We can attempt to understand these systems and their responses to perturbations using computational models (green) of varying complexity.

As a field, our scientific investigations often seek to understand a complex system in light of perturbations to the system’s structure or function. Such perturbations may be enacted by the environment, designed by laboratory personnel, or offered by trained clinicians. The systems upon which these perturbations impinge are myriad, but our remarks will largely center around the systems of brain, symptom, and behavior (Fig. 1). In what follows, we will use the term brain to refer to the cerebrum and all of its components; the term symptom to refer to a self-reported experience that may be especially intense in states of mental illness; and the term behavior to refer to a measurable (and measured) action that need not be altered in psychiatric or neurological disorders. Below we describe the components of each system and their interconnected nature, as well as the relevance of interconnectivity for perturbations. This discussion will allow us to move to a description of associated modeling efforts in the following sections.

## The neural system

### Parts and relations

Neural systems are composed of computational and modulatory units that serve to receive, process, alter, and transmit information. Such units exist across multiple spatial scales, from the microscale of individual cells to the mesoscale of cortical columns, and thereafter to the macroscale of nuclei and cytoarchitectonically distinct areas [1, 2]. Independent of the spatial scale, units are interconnected with one another in a heterogeneous pattern of physical links that support communication, coherence, and other sorts of functional interactions [3–5]. Physical links at the small scale comprise synapses and gap junctions [6], and at the large scale comprise large axonal bundles or white matter tracts [7–9]. The complete neural system thus comprises both parts and pathways, or conduits for relations.

### Network representation

Early studies of macaque, cat, and *Caenorhabditis elegans* connectomes suggested the potential utility of representing neural systems as formal mathematical objects known as graphs or networks [10]. In its simplest instantiation, a network is composed of nodes which represent a system’s units, and those nodes are connected in pairs by so-called edges which represent a system’s inter-unit links, relations, or interactions [2]. From the pattern of edges between nodes, the structure, function, and dynamics of systems can in part be inferred, offering putative explanations for a system’s manifest complexity [11]. The network representation has since proven useful in characterizing the interconnected architecture of neural systems across many organisms including humans [12], and the alteration of that architecture in conditions of mental illness and injury [13–15].

### Network perturbations

The notion that neural systems are fundamentally network systems has changed the way we think about clinical interventions to support mental health. The most common mechanism of action of pharmacologic interventions for neuropsychiatric illness is the manipulation of distributed neurotransmitter systems. Neurotransmitter systems fall into two major categories: fast ionotropic neurotransmission and slow metabotropic neurotransmission [16]. The latter includes the frequently targeted serotonin [17–19], dopamine [20], and norepinephrine [17, 18] systems. Metabotropic neurotransmitters modulate ionotropic neurotransmission [21, 22], and as a result, the effects of perturbing neurotransmission unfold over multiple time scales. Varying metabotropic neurotransmitter input, also called neuromodulation, can drive groups of neurons to exhibit different patterns of coordinated firing [21], suggesting that neurotransmitters fundamentally influence network interactions rather than eliciting simple increases or decreases in firing.

Neuromodulatory medications cross the blood–brain barrier to reach every brain region, with effects depending on binding profiles of drugs as well as regional expression of neurotransmitter receptors [23]. The mechanism of neurotransmitter action also differs across the brain and depends on external demands [22, 24]. This spatial heterogeneity and state-dependence prevents us from understanding how pharmacotherapy perturbs individual regions in isolation, because a region of interest may be affected by changing inputs from its interacting partners. In our analogy, when there are winds coming from several directions, it is similarly hard to tell which gust had the biggest impact on the tree. Network representations resolve this complexity by allowing the brain to be studied as a whole, rather than as individual regions or even pairwise interactions. Indeed, manipulations of neurotransmitters shift interregional interactions in a way that can be parsimoniously characterized as affecting global network flexibility, segregation, and integration [25, 26]. Metabotropic neurotransmitter systems may contribute to separable components of large-scale fluctuations in cortical activity [27], which may allow the design of future therapeutics to target specific subsets of network interactions. Dynamical network models of neural systems provide a promising computational toolkit for testing hypotheses about the associations between neurotransmitter systems, brain activity, and behavior.

## The symptom system

### Parts and relations

Symptoms of mental disorders tend to co-occur. For example, individuals receiving treatment for psychiatric disorders experiencing sad mood are likely to also experience anhedonia [28]. Indeed, the formation of symptom clusters is reflected in diagnostic practices; a patient must present with five (or more) symptoms out of a list of nine to be diagnosed with major depressive disorder according to the Diagnostics and Statistical Manual [29]. Traditionally, this symptom co-occurrence is explained by assuming that an underlying, latent disease gives rise to groups of symptoms.

### Network representation

Network perspectives of mental disorders provide an alternative account to explain symptom co-occurrence [30, 31]. A network approach to psychopathology suggests that symptoms are not simply interchangeable indicators of a latent disorder. Instead, individual symptoms are interesting in their own right and influence one another over time, forming causally connected networks [32, 33]. In line with this perspective, individual symptoms of disorders can vary in their time of onset, their severity, and even in their response to treatments [34–37]. Further, there exists a substantial body of literature that makes use of intensive repeated measures of symptom intensity from moment-to-moment and day-to-day to quantify time-lagged associations between symptoms; these studies provide strong evidence for symptom interplay across time [38–40].

### Network perturbations

The network perspective provides a framework within which to capture the importance of individual symptoms as well as the intuitive notion that symptoms act to causally influence one another across time. By focusing on individual symptoms and their interplay, it also puts forward a new perspective for understanding the etiology of mental disorders. A key role is given to a phenomenon termed hysteresis [30, 41] whereby once a system shifts to an alternative state (e.g., a person becomes depressed), it tends to remain in the new state until the external input that led to the change is changed back to a much lower level than was needed to trigger the change in state. The maintenance of a state of psychopathology, consisting of high levels of symptom activity is theorized to occur only in strongly connected networks, consisting of many time-lagged associations between network components. When a symptom in such a strongly connected network is perturbed (e.g., the experience of a stressful life event), symptom activity spreads through causal associations in the network to other symptoms, resulting in a self-sustaining symptom network.

### Beyond symptoms

One of the early principles of the network theory of mental disorders was that the components or symptoms in a psychopathology network correspond to problems that have been codified as symptoms in the past century and that are detailed in contemporary diagnostic manuals [30]. This focus on symptoms encoded in diagnostic manuals as the main components of a symptom network preserves the rich information available in diagnostic manuals, emerging from years of clinical observation [42]. Working from this principle, other aspects of human functioning not encoded in diagnostic manuals must (i) constitute a symptom (e.g., a brain-based realization of a symptom), (ii) constitute a symptom-symptom connection, or (iii) act as a variable in the external field that causes a symptom.

## The behavior system

### Parts and relations

What are these extra-symptom aspects of human functioning? It may be reasonable to refer to some such aspects as behavior. Informed by the scientific method, one might define a behavior as a measurable (and measured) action: an interaction with the environment. A common example is a reaction time measured in response to a cognitive demand. A second kind of an extra-symptom aspect of human functioning is a person’s emotional state. While distinct from behavior, emotional states can similarly be measured in response to a task manipulation. Both states and actions, however, are not independent but instead can interact. Humans may be more likely to move from a state of contempt to a state of fear than from a state of disgust to a state of joy [43]. Similarly, the measured speed of an action is typically inversely correlated with the measured accuracy of an action [44, 45]. Moreover, a given action might impact a subsequent action manifesting as an asymmetric switch cost [46–49]. Thus, both behavior and state are systems composed of units (actions; states) and their relations (either correlative or causal). This distinction we make between behavior and symptoms is practically useful, as we will see throughout this review; yet, in truth the two exist along a clear continuum of human experience.

### Network representation

The network perspective so relevant for psychopathology can also be easily extended to behavior and state systems more broadly. Indeed, theorists, especially developmental scientists, have long considered persons as complex systems, with feelings, thoughts, and actions that are interconnected and that change over time [50–52]. In applications beyond mental disorders, networks consisting of emotions, social behaviors, and other psychological states have been created to realize this notion of persons as complex systems. In studies of non-human animals, recent work has focused on behavior alone, algorithmically coding extensive video footage and separating actions into behavioral units (network nodes) and the likelihood of transitioning between such units (edges) [53, 54]. Collectively, such network maps underscore the fact that domains of function are not binary or unitary. Instead, they are composed of a collection of units whose interactions give rise to the emergent modes of being [55].

### Network perturbations

Human behavior is commonly impacted by changes in our environment or the particular social context in which we find ourselves [56–58]. For example, in a stressful circumstance the probability of transitioning from dinner to a drink may be high, whereas in a non-stressful circumstance the probability of transitioning from dinner to a run (or a good book) may be high. Understanding how perturbations to the environment or context lead to predictable changes in a complex system of interacting behavioral units could have far reaching consequences for social policy [57], workplace standards [58], and the practices of educational institutions [56]. Moreover, such an understanding could inform the development of environment interventions for patients with mental illness, consistent with recent work using theatre improvisation training as a recovery-oriented intervention for youths at clinical risk for psychosis [59].

## Shared modeling needs across the three systems

Across the three systems described above—brain, symptom, and behavior—there are many shared features that could be taken into account by models. First, all three systems are composed of parts that can be perturbed by external factors. Second, perturbations to one part of the system can impact neighboring parts. Third, a system’s response to a perturbation may depend not only on the part and its neighbors but also on the relations among neighbors and the broader network. When we choose a modeling approach, we often implicitly choose which of these three facts we will acknowledge and investigate.

Like the tree in the wind, our models range from trunk, to trunk with roots, to the entire forest. For example, a commonly applied form of the GLM is used to assess how external perturbations affect the system’s part(s). An impulse response model is used to assess how an external perturbation affects a given part of a system and travels into the network through that part’s connections. Finally, a network control model is used to assess how an intrinsic or exogenous perturbation can be designed to drive a desired network-wide response. In the sections that follow, we describe each of these modeling approaches, review their use in studying brain, symptom, and behavior, and evaluate their relative strengths and weaknesses. This discussion will allow us to move to a description of future directions in the following section.

## Modeling the tree as simply a trunk

**Fig. 2:**
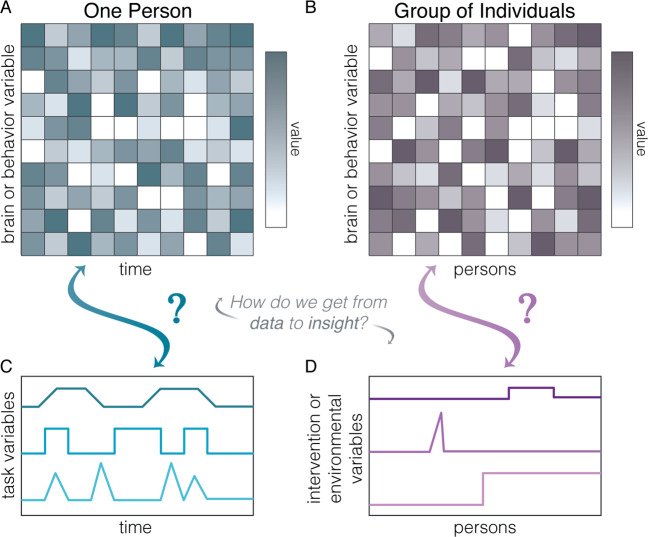
Univariate or multivariate models to evaluate the effects of perturbations on system parts. **a** The systems that we study are often time-varying over multiple scales. **b** Some systems that we study are composed of canonical trait-like variables that vary across persons, possibly ordered by severity of condition, age, or even an identification number. How do we take these data and from them extract insight? **c** We can understand the impact of task variables on systems like those displayed in panel **a** using several approaches including a typical form of a general linear model. **d** We can also understand the impact of interventions or environmental variables on systems like those displayed in panel **b** using a similar general linear model.

We will begin with one of the simplest and yet arguably most pervasive approaches to modeling complex systems that are perturbed by external variables. Specifically, we will consider a commonly applied form of a GLM that is typically used to evaluate the effect of an external variable (e.g., from the environment) on the activity or expression of parts of a system. The underlying assumption of this approach is that the system is composed of parts that can be perturbed (Fig. 2). Perhaps we have measured brain or behavior variables over time (Fig. 2a) and wish to understand how the perturbative drives of a task’s structure affect those variables (Fig. 2c). Or perhaps we have measured brain or behavioral variables over persons (Fig. 2b) and wish to understand how the perturbative drives of interventions or environmental events affect those variables (Fig. 2d).

A rather simple form of a GLM allows us to answer these questions in a mathematically rigorous way with appropriate inferential statistics [60]. For a formal mathematical treatment, see for example [61, 62]. In general, these models take the form **Y** = **Xβ** + **ε**, and in this context, **Y** is a vector measuring a perturbed variable over time, **X** is a matrix measuring the magnitude of one or more perturbations over time, **β** is a vector of coefficients that estimate the static effect of each perturbation, and **ε** is a vector of additive errors. In order to identify a unique solution to this equation, there must be more observations (rows) than the number of columns in **X**, and no two columns in **X** can be linearly dependent, though regularization methods can help to overcome this limitation [62].

**Fig. 3:**
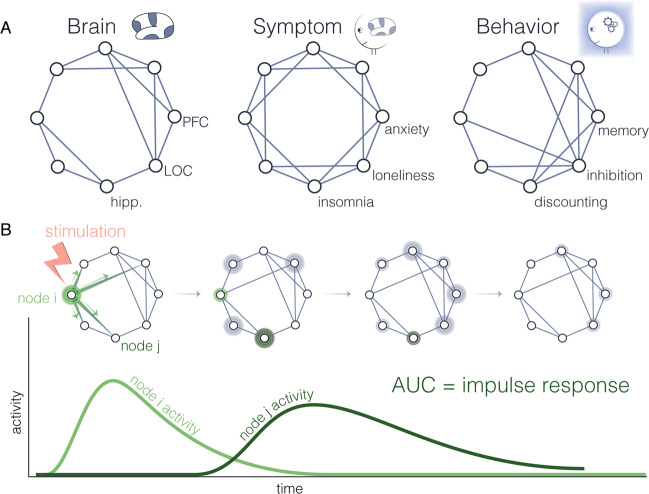
Impulse response models. **a** (Left) A brain network encoding across-time or across-person relations between regions. (Middle) A symptom network encoding across-time or across-person relations between symptoms. (Right) A behavior network encoding across-time or across-person relations between cognitive, behavioral, or neuropsychological variables. **b** Illustration of the response of a network to an impulse (pink). The network response is shown across the top row; the bottom plot shows the response of the stimulated node (light green) and the response of a second node (dark green). The area under the curve is an impulse response metric.

**Fig. 4:**
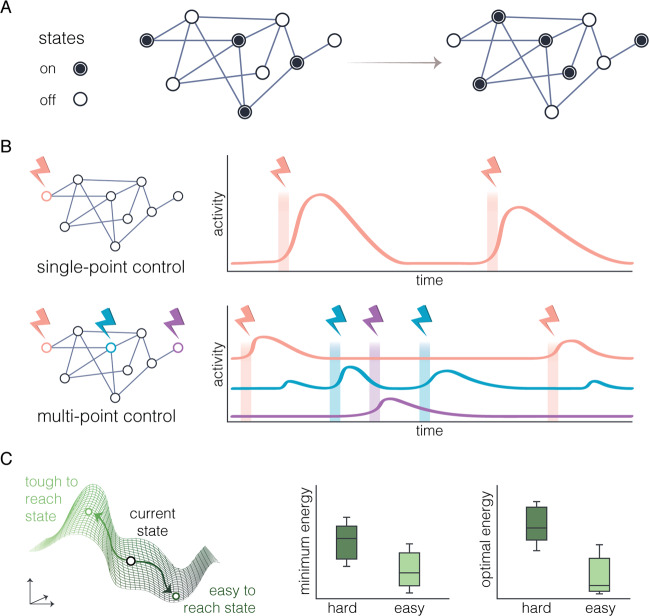
Network control models. **a** Network control theory asks how to design interventions that move a system from one state (values on nodes) to another state (different values on nodes), by constraining the cause of state change to occur along edges. **b** Interventions can be enacted in a time-dependent manner from a single region (top) or from multiple regions (bottom). **c** (Left) Illustration of an energy landscape that is dictated by the architecture of the network and that in turn encodes the fact that some transitions are easier to drive than others. (Right) Two complementary metrics that quantify the ease of a state transition.

### Utility in neural, symptom, or behavior systems

To make knowledgeable interventions for the full human, ideally we would understand the relations among all three systems (brain, behavior, and symptoms) in detail. Yet, for practical reasons, many of our empirical studies are constrained to only one part of this multi-system organism. Here (and in the next few sections) we will therefore consider how each system is modeled, and return to their interdigitation towards the end of the review. We do acknowledge, however, that fundamental progress in neuropsychopharmacology will require us to understand how all three systems interact with one another.

### Neural system

GLMs are frequently used in the analysis of task functional magnetic resonance imaging data to identify the neural correlates of cognitive functions. This inference is achieved by measuring the BOLD signal change elicited by a particular stimulus, or contrast between stimuli [61]. Classically, one assumes that a neural event elicits a slow increase in BOLD activity, defined by a sum of gamma functions known as the canonical hemodynamic response function. A GLM is used to estimate the height of this increase independently at every voxel or brain region, thus inferring the extent to which individual regions engage in specific cognitive processes. Although many different types of GLMs can be specified, the most commonly used type does not assume any interactions between the system’s parts.

The use of GLMs has helped to localize neural representations of stimulus attributes and identify areas involved in task-general and task-specific cognitive processes. Early work revealed that the fusiform gyrus exhibited apparently selective activation to images of faces. Other studies used GLMs to demonstrate activation of the amygdala and ventromedial prefrontal cortex while processing emotional salience of images [63]. Cognitively demanding tasks were found to elicit deactivation of posterior cingulate and medial prefrontal cortex with activation of dorsolateral prefrontal cortex [64], now known as the default mode system and frontoparietal system, respectively. These two opposing systems were subsequently identified from spontaneous blood oxygen level dependent (BOLD) signal fluctuations observed in resting state fMRI [65], suggesting that the signatures of task activation are etched into the intrinsic network architecture of the brain. Interestingly, while the default mode system is deactivated across many tasks, GLM-based studies have also revealed that default mode activity increases during tasks requiring autobiographical memory and theory of mind [66]. GLMs have provided critical insights into multiple cognitive processes, including but not limited to primary stimulus processing, internally- and externally directed attention, and self-referential thought.

In addition to revealing cognitive processes associated with brain areas, GLMs have served as a foundation for the subsequent development of more complex analytic techniques. For instance, GLMs are so commonly used in fMRI research that publicly available tools exist that allow for meta-analysis of more than 500,000 activation maps from over 14,000 studies [67], facilitating additional discoveries. Early GLM studies also laid the framework for the task fMRI protocols of large sample size studies such as the Human Connectome Project [68] and the Philadelphia Neurodevelopmental Cohort [69], which have subsequently revealed a great deal about predicting individual differences in cognition from brain activation [70–72]. Overall, GLM techniques have both revealed important associations between cognition and brain activation, and further motivated a rich literature on the neural substrates of individual differences in behavior.

### Symptom and behavior systems

The GLM has been the workhorse of the psychologist’s statistical toolbox when examining how mental illness, and behavior more generally, is impacted by changes in the environment or is targeted during intervention efforts. Examples relevant to neuropsychopharmacology are plentiful and include tests of the effects of behavioral interventions designed to reduce major depressive disorder [73], the effects of medication on nicotine withdrawal during smoking cessation attempts [74], and the effects of stressful life events on cognition [75]. GLM approaches treat other symptoms and behaviors that could plausibly affect the outcome variable of most interest as control variables. This approach is taken to identify, as cleanly as possible, the effect of the intervention or event of interest on the symptom or behavior of interest, independent of other possible sources of influence. Thus, the potentially interconnected nature of the symptoms of a disorder or the sets of behaviors within an organism’s repertoire is typically ignored or viewed as a nuisance to be accounted for during statistical modeling rather than an attribute of interest.

### Relative strengths and weaknesses

Restricting our attention to neural systems alone, it is worth mentioning a few strengths and limitations of the commonly used form of the GLM. While this model has been highly useful for relating regional activation to cognition, it is nevertheless a mass univariate test as applied to fMRI data. As a result, the model is agnostic to the role of interactions between brain areas in cognition. This common form of the GLM and other trial-averaged measures may therefore fail to detect spatiotemporal waves of activation by virtue of modeling each region or voxel independently [76]. Additionally, traditional approaches for GLMs involve the assumption that neural activity in all brain areas and all individuals under study exhibit the same hemodynamic response profile, which is unlikely to be the case [77–79]. Issues of hemodynamic response variability aside, the field has continued to debate the appropriate method for assessing statistical significance of brain activation maps for almost two decades since the introduction of the technique [61, 80, 81]. For clinical use, resting state connectivity measures may indeed provide more reliability than task fMRI and the commonly applied form of the GLM, depending on the extent to which a lack of cognitive engagement in certain clinical groups acts as a confound [82].

Broadening out from neural systems alone, several relative strengths and weaknesses of the commonly used form of the GLM are shared across systems. A shared strength is that the approach can flexibly address univariate and multivariate processes; that is, it can go beyond the investigation of a single part of the system to acknowledge the fractionated, particulate, and decomposable nature of complex systems [83]. A shared weakness of the typical model is that it does not formally account for the embeddedness of parts in a complex whole; in other words, perturbations are assumed to affect the system’s parts, but not in a way that depends systematically on neighboring parts. This limitation motivates the use of alternative models with a greater level of complexity. In the next section, we explore just such a model type, the impulse response model (Fig. 3), which serves to address the shared limitation of the typical GLM. Alternative models that we do not describe in this review include those that assess the statistical relationships between (i) a measure of connectivity between two regions and (ii) a measure of symptoms or behavior. Particularly good examples of such models include psychophysiological interaction, partial least squares, and canonical correlation analysis [84, 85].

## Acknowledging the tree’s root system

Unlike the commonly used GLM just described, network models explicitly encode the relations among a system’s parts. Network models of brain, symptoms, and behavior provide insight into which set of system variables contains information that improves the prediction of another set of system variables. The models provide information about the individual associations among network nodes. Yet, in many applications, interest goes beyond this edge-central representation and towards an examination of how the system behaves as a whole. Capturing system-level functioning necessitates a tool capable of modeling the interplay between network units in a high-dimensional system.

Early efforts to capture such system-level notions focused on node centrality and network density. A common measure of node centrality is degree centrality, defined as the number of edges emanating from a node [86]. Here we will frame our discussion within the context of symptom systems, and broaden to other systems later. Node centrality has been identified as a potential index for interrogating networks to find symptoms that would facilitate the spread of behavior through the network, activating self-perpetuating sequences of symptoms across time [87, 88]. The complementary notion of density is defined as the fraction of possible edges that exist in a network. Network density has similarly been identified as a potential index for characterizing the likelihood that a network will support reverberating symptom activation [89, 90]. The process is analogous to the behavior of two sets of domino tiles: one in which the tiles are far apart and a second in which the tiles are close together. In a densely connected symptom network, when one tile falls it causes other tiles to topple and activity ripples through the system [91]. The use of both centrality and density are limited in their focus on direct connections, containing little information about how symptoms might affect each other indirectly as activity flows through the entire network [92].

Recent efforts have turned to impulse response analysis (see ref. [93, p. 51] for general mathematical treatment and Blaauw et al. [94] for treatment specific to symptom and behavior networks). Using a network model of the interactions between variables across time, impulse response analysis involves first simulating an instantaneous, exogenous impulse (referred to as a shock) to certain variables in the network. Then, one charts how this impulse propagates through the network, along the time-lagged edges. The impulse response function shows the hypothetical change in a variable in response to a simulated increase in one of the other variables over a horizon of several time points. Crucially, the activity in one node observed after a shock to another node in the system contains system-level information. The impulse felt by the non-shocked node is due solely to the flow of activation along the network’s edges originally emanating from the shocked node. In this manner, impulse response models (unlike the commonly used form of the GLM) acknowledge the fact that a system’s parts influence one another; moreover, that precise influence constrains the response of the system to a perturbation.

### Utility in neural, symptom, or behavior systems

#### Symptom and behavior systems

Despite their marked advantages over simpler models, impulse response models have not yet been used extensively in the context of behavior and symptom systems. Notable exceptions are studies of depression [95], anxiety [96], and tobacco dependence [97]. Here we review each in turn before moving to a discussion of the use of impulse response models in neural systems.

In a sample of subclinically depressed individuals, Bos et al. [95] examined affect dynamics in daily life, and the extent to which physical activity and experiences of stress impacted affect in participants with anhedonic symptoms versus those without. By completing affect, behavior, and cognition items via an electronic diary three times a day for 30 consecutive days, participants generated time series that were then subjected to vector autoregression (VAR) in which each variable was regressed on its own lagged values as well as the lagged values of the other variables. This process resulted in, for each participant, a network indicating the time-lagged associations between six variables: high-arousal positive affect, low-arousal positive affect, high-arousal negative affect, low-arousal negative affect, stress, and physical activity. Impulse response analysis was then applied to these VAR models to examine the impact of physical activity and stress on affect over a horizon of ten time points. When an increase in physical activity was simulated, the other variables only marginally changed in response. When an increase in stress was simulated, affect in non-anhedonic individuals showed a stronger increase relative to affect in anhedonic individuals. These findings ran contrary to expectations, highlighting the surprising findings that can emerge when the flow of activity from one behavior to another is allowed to play out in a complex, high-dimensional system.

In the preceding example, the effect of an individual behavior (e.g., physical activity) on an individual outcome (e.g., negative affect) was examined. By using impulse response analysis, the observed association between physical activity and negative affect took into account the broader system consisting of six variables. The effect of physical activity on negative affect consisted of the effect of a simulated increase in physical activity filtering through direct pathways to impact negative affect, but also via indirect pathways emerging from the complex interplay between physical activity and negative affect with other variables in the system (e.g., stress) across time. Other uses of impulse response analysis in the literature have focused on capturing system properties occurring at a higher level of topological analysis [1]. Using experience-sampling data assessing six symptoms of tobacco withdrawal (anxiety, craving, depressed mood, irritability, hunger, and difficulty concentrating) in daily smokers during a 2-week smoking cessation attempt, Lydon-Staley et al. [97] examined the effects of cessation treatments on the reverberation of symptom activity across time. After constructing person-specific networks indicating the time-lagged associations between symptoms, impulse response analysis was applied to quantify how a simulated increase in one symptom affected other symptoms. Specifically, the time profile of a symptom of interest following perturbation of another symptom was simulated over 100 time steps. The time profile was then examined to identify the recovery time of the symptom, quantified as the number of time steps from perturbation to equilibrium (see also ref. [98]). The average time taken for a symptom to return to equilibrium after perturbation was faster in participants in an intensive combination treatment condition relative to participants in a matched placebo condition. Broadly, the study demonstrates the distinct insights that an impulse response analysis can provide on the question of how interventions can differentially impact the dynamic interplay between symptoms.

The preceding examples used self-reports of behavior, symptoms, and various states as network components and employed impulse response analysis to capture the effect of a change in a variable of interest (e.g., physical activity) on an outcome of interest [95] (e.g., affect) or to capture the system-level notion of hysteresis [97, 98]. In a striking example demonstrating the flexibility of both the network approach and impulse response analysis, Yang et al. [96] used a combination of the two to capture adolescent emotion system dynamics. Second-by-second psychophysiological time series, consisting of respiratory sinus arrhythmia (an indicator of parasympathetic nervous system activity [99]) and skin conductance level (an indicator of sympathetic nervous system activity [100]), and second-by-second self-report of distress measured using video-mediated recall were collected from 130 adolescents. In constructing a network of the time-lagged associations embedded in this tri-node system, notions of emotional concordance and emotion regulation were captured. Emotional concordance is the synchronized combination of psychophysiological, cognitive, and behavioral components of the emotion system [101]. Emotion regulation is the ability to produce a flexible, controlled emotional response to environmental events [102]. An impulse was given to each node separately and, for each simulation, changes in all three nodes were tracked until activity reached equilibrium. The value of node activity at this equilibrium level provided an index of system reactivity. In contrast to prior studies, the approach utilized here strongly underscores the interrelated nature of emotional states.

### Neural system

Impulse response models and related techniques have also been applied to the study of neural systems. Early fMRI studies sought to characterize the impulse responses of primary visual cortex to single stimuli [103, 104], without estimating an underlying multicomponent network model. More recent studies have used activity flow, a variant of impulse response models, to estimate task activations from activity spread along the whole-brain resting state functional connectome [105, 106]. Work from this group has focused on average controllability, which quantifies the area under the curve of the impulse response of each brain region, using linear dynamics along structural networks obtained through diffusion-weighted imaging [107, 108]. Average controllability of frontolimbic regions increases with age [109, 110] is associated with impulsivity [110], and is reduced in patients with longstanding bipolar disorder [111]. Studying the effects of local perturbations in nonlinear systems [112, 113] is promising due to their ability to recapitulate oscillations characteristic of brain signals [114]. However, a validated toolbox of regional measures of impulse response does not exist for such systems, though one study suggests that linear impulse response metrics may extend to nonlinear systems [115]. These findings suggest that impulse response metrics in neural systems capture behaviorally relevant information that may serve as useful biomarkers and theoretical testbeds for treatments in the future.

### Relative strengths and weaknesses

Impulse response analysis provides a fitting analytic match to theories emphasizing the spread of activity through a network of connected components. It incorporates both the directionality and the sign of edges during computation, allowing insight into complex features such as positive and negative feedback loops. This sensitivity stands in contrast to approaches that take the absolute value of edges in brain or behavior networks before computing the density of a system to quantify the potential for activity spread through a network [89, 90] or those that decompose strength into in-strength and out-strength when identifying influential nodes [39, 116].

Alongside these strengths, a number of limitations are important to consider. In using impulse response analysis coupled with a VAR model, we make the assumption that the system and the interactions among units in the system are time invariant. It is this assumption that allows us to forecast how the system will react to a perturbation. Time-varying VAR models may be appropriate in cases in which system dynamics change over time [117] and would limit the use of impulse response analysis for predicting system activity following perturbation to periods of time where time-invariant VAR models provide a sufficient description of system dynamics. A related consideration associated with limiting one’s focus to periods during which time-invariant processes may be assumed is that densely sampled time series data are often required to fit VAR models. Guidelines for the amount of data required remain to be established, will depend on the size of the system one is attempting to model, and will benefit from the transparent reporting of when time series length emerges as a barrier to VAR modeling (see ref. [98] for an example of such reporting). An additional consideration concerns the system units used to build a model upon which we simulate perturbation. The results of simulated perturbations will differ depending on the units we assume are relevant to a system. Yet, for even the most intensively studied psychological disorders (e.g., major depression) there exists substantial heterogeneity across measurement instruments as to which symptoms are relevant [118]. We note that these considerations are not specific to impulse response analysis but to network approaches more generally.

A consideration more specific to impulse response analysis relates to the time horizon chosen to examine system responses to perturbation. The examples of impulse response analysis in brain, behavior, and symptom networks described above flexibly used several different time horizons. For example, one used a time horizon of 10 time points following perturbation [95]; one used a backward search to identify the time taken for the system to return close to its starting value following perturbation [97]; and another identified the time at which the system settled at a point of equilibrium [96]. This difference across studies highlights the flexibility with which impulse response analysis can be used to capture the system responses of most interest. Yet, it also reveals a limitation of the method: chiefly, the difficulty of incorporating information about particular system end states that may be of theoretical interest. In the next section, we therefore turn to a model that addresses this limitation: namely, the network control model (Fig. 4).

## Expanding to the forest

Network control models stipulate how the state of a system changes over time as a function of intrinsic dynamics and time-varying external inputs. Note that the impulse response model is a special case of network control models, which captures how the system evolves following a single initial input. Like impulse response models, network control models define a system of *N* interacting units, each of which has some measured property at time *t* represented in a vector **x**(*t*). For example, the *N* units may represent behavioral symptoms, neurons, or mesoscale brain structures. The values of **x**(*t*) might therefore contain measures of symptom intensity, firing rate, or BOLD fMRI signal, respectively. For a formal mathematical treatment, see [119].

Distinct from the special case of impulse response models, a generic network control model may contain time-varying external inputs into the system represented by the vector **u**(*t*). These inputs are filtered into the units and dimensions of **x** via some transformation. The temporal progression of the system can then be described as $\big\lceil\!{\dot {\boldsymbol{x}}}$(*t*) = *f*(**x**, **u**, *t*). This function posits that the change in state variables ${\big\lceil\!{\dot {\boldsymbol{x}}}}$(*t*) is determined by some function of the current state of the system, intrinsic interactions between the *N* units, and filtered external inputs. In the case where **x**(*t*) represents symptoms, **u** might represent an adverse life event, which, when filtered by some function, has a characteristic impact on the symptoms in **x**(*t*). In the case where **x**(*t*) represents neural activity, **u** might represent the binding affinity of a drug, which has a characteristic impact on neural activity when filtered through spatial patterns of receptor expression. Network control models are particularly well-suited to the study of neural, symptom, and behavioral systems because they capture the continuous interplay between intrinsic dynamics and the external environment [120].

### Utility in neural, symptom, or behavior systems

#### Neural system

Numerous studies have applied models of network control to mesoscale [111, 121–124] and microscale [125–127] neural systems. Due to analytical and computational limitations in the study of nonlinear systems, most studies have used linear, time-invariant network models [128]. The most common form is $\big\lceil\!{\dot{\boldsymbol{x}}}$(*t*) = **Ax**(*t*) + **Bu**(*t*), where **A** contains a set of measured connections between *N* neural units and **B** is a linear input filter. A subdomain of network control theory, known as optimal control, allows one to solve for the external inputs **u**(*t*) needed to drive a linear system **A** from a specified initial state to some desired target state [121–123]. The literature on optimal control is related to a sister literature on reinforcement learning and other continuous action policy search methods [129].

Using the control framework, the latent inputs driving brain activity across multiple cognitive tasks can be recovered [130, 131]. Data-driven network control models have been used to successfully decode mood fluctuations [132] and motor patterns underlying speech [133] from implanted electrode grids in patients with intractable epilepsy. A combination of open loop and optimal control techniques can explain how exogenous electrical stimulation drives the brain into activity states favorable for episodic memory recall [123]. Patients with schizophrenia were found to theoretically require stronger inputs to reach whole-brain fMRI activation patterns associated with working memory performance, while the strength of inputs required to maintain those states varied with estimated prefrontal D1 and D2 receptor expression [134]. Patients with chromosome 22q11.2 deletion syndrome, which confers genetic risk for schizophrenia, were found to spend more time in fMRI activation patterns requiring stronger inputs to maintain [135]. These initial studies lay the framework for merging neurotransmitter pharmacology and brain stimulation approaches with network control models of neural activity.

### Symptom and behavior systems

Mental health professionals are increasingly interested in interrogating personalized behavior networks (broadly encompassing emotions, cognitive processes, symptoms, and actions) in order to inform treatment [136]. A proposed use of the estimated networks is the identification of nodes that drive the behavior of other nodes and that, thus, can be targeted early in treatment to arrive at a desired system state [137, 138]. Methods of identifying nodes with particularly strong downstream effects on other system nodes include patient–provider discussions of estimated networks to identify potential causal chains [138] and clinician examination of edges in estimated networks to identify edges with particularly large coefficients [137]. Nodes with relatively many and relatively strong out-going edges (high out-strength in the parlance of graph theory) are also thought to be important nodes for influencing other network nodes [139].

Network control models have recently been extended to psychological networks [140] to provide a statistical framework capable of guiding personalized interventions. Data-driven control models that have been applied to neural systems to explain how exogenous stimulation drives the brain into desired activity states [123] can be readily translated to symptom and behavior networks to identify optimal treatment targets and to simulate the theoretical efficacy of potential interventions. The translation of network control models from neural systems to symptom and behavioral networks is an important step with promising potential to overcome the limitations of relying on visual inspection and estimation of centrality indices of symptom and behavior networks to guide treatment.

### Relative strengths and weaknesses

The mathematical sophistication of the network control models provides an elegant simplicity and validity in tackling the true complexity of neural systems and behaviors. Of course, we must always acknowledge that although more complex models may more closely align with the complexity of the true system, they can also hamper intuitions and be difficult to interpret as well as fit to data. Indeed, the practical clinical utility of network control models has yet to be realized due to this type of limitation. For example, the functions relating the state of the system and the inputs are often unknown in practice. In the case of neurotransmitter systems, this ignorance stems from an inability to link molecular and neuronal mechanisms with mesoscale measurements of brain activity. In the case of behavioral symptoms, it is unknown how life events reliably impact sets of symptoms over time. Studies of network control [123, 124, 134] have gleaned useful insights by solving for unfiltered, theoretical inputs that act directly upon the *N* units of x(*t*). However, practical utility depends upon the link between real interventions (i.e., pharmacologic agents or brain stimulation) and brain state (i.e., x(*t*)). Data-driven approaches may prove useful for approximating these link functions as well as the underlying network interactions [130, 141, 142], but they will require careful validation with large amounts of data from single individuals before they can be used in the clinic. Gleaning interpretable and intuitive clinical decision support from complex models may require clinicians to have independent expertise, likely limiting their utility to highly specialized care settings.

From a practical point of view, the utility of network control models depends upon the certainty with which the network itself can be estimated, and the verity of the form of dynamics used in the model. When using network control models in the context of human structural brain networks, care should be taken to consider the impact of distinct diffusion imaging sequences that differ in their spatial resolution, scan duration, and number of diffusion directions sampled. Similarly, when using network control models in the context of symptom and behavior networks, formal tools to quantify uncertainty on each of the network edges linking symptoms (or linking behaviors) will be important. We recognize that thorough methodological studies have yet to be done to define clear benchmarks for the amount and type of data needed to build accurate models to ensure a given level of confidence in statistical inference. We look forward to future work addressing these important issues.

## Future directions

The three modeling approaches that we have discussed here are motivated by the overarching goal of understanding how a network system (brain, symptom, behavior) responds to a beneficial or maleficent perturbation (Fig. 1). How does the tree bend in the wind? Each model type seeks that understanding by implementing a different level of complexity in the analytical assumptions and model formulation: the most commonly applied form of the GLM (tree as trunk), impulse response models (tree as trunk with roots), and network control models (forest with interdigitated root system). Each new model advances the state of the field by addressing the limitations of the previous model. While the process of model development and selection continues apace, many opportunities exist to take full advantage of the approaches available today.

In the realm of basic science, there is one troublesome question left open by our entire discussion. Precisely how are the three systems interlinked with one another? How exactly does one system impinge upon or support another? And even more: How can such interlinkage be modeled? The pines of Kaliningrad Oblast thrive in a broader ecosystem alongside diverse other flora. How might the approaches that we review be extended to deal with the presence of more than one system, with its own notion of a part (or network node) and its own notion of a relation (or network edge)? Might we need an entirely new approach all together? Perhaps it could prove useful to consider canonical correlation analysis as a means of deriving dependencies between two systems [143, 144]. Such dependencies could be used to develop multilayer network models of two or more systems [145], which in turn could be studied with the principles of network control [146]. Are such possibilities reasonable next steps? Or are more foundational studies needed first?

In considering clinical translation, perhaps the most critical challenge we face is understanding and parsing heterogeneity [147]. Differences in participant, treatment, and outcome characteristics challenge the development of experiments, the collection of necessary data, and the subsequent inferences that can be drawn. Implicit in understanding heterogeneity is understanding the time scales over which participant characteristics (brain, symptom, and behavior) are trait-like versus state-like, as this distinction can clarify whether differences are true or only apparent. Traits are often hailed as the gold standard in clinical medicine due to their reproducibility; that is, the fact that they remain unchanged over iterative measurement. Yet, complex systems typically display rich temporal dynamics in which the reproducible feature is a dynamical rule (fit by first, second, and sometimes even third derivatives) rather than by any single snapshot of variable values. By requiring reproducibility of a static measurement, rather than conscientiously seeking reproducible dynamical trajectories through a rich state space, are we hamstrung in truly understanding the intricacies of mental illness? What are the appropriate time scales over which to be measuring (and by extension modeling) these systems? And are those time scales the same as or different from the appropriate time scales over which to be measuring the effects of perturbations? How would the answers to these questions change the way we model?

Beyond basic and clinical science, the methodologists might ask whether statistical models (such as those described here) are truly the workhorse of the future. Will statistical models eventually be more formally complemented by machine learning approaches and if so, how? It is true that some machine learning models have been criticized for lack of interpretability, and others for bias and lack of fairness in their respective algorithms [148, 149]. Yet, it is important to acknowledge that some computational models can also lack interpretability. In determining the appropriate balance between statistical models and machine learning models, it is important to clearly state the sort of understanding the investigator wishes to obtain. A black-box algorithm may be superior if the question is predicting treatment outcome based on a complex biological measure, but will do less well if the goal is to understand the computational role a specific brain region plays in a given behavior. Computational models also have the benefit of being written in the same language as generalizable formal theories, which in turn are structured in a way that humans can naturally reason about and with. A complementarity between statistical models and machine learning could take on several forms. Theory-agnostic algorithms may prove useful for making a first reasonable estimate of appropriate model parameters, or perhaps for determining which parts of a many-part system must be included in the model to canvas the space of possible dynamics appropriately. Future work is likely to better formalize the types of interdigitation between algorithm and model that may best advance the science toward our collective goals.

A key consideration in linking theory, modeling, and experiment is the utilization of an appropriate framework for causal inference [150]. Our discussion has focused on studying and discovering the effect of an intervention in a complex system. This effort can quite naturally lend itself to the identification of causes and mechanisms. The three methods we cover differ in their explicit account of causality. For example, the particular form of GLMs that we discuss seeks to measure the statistical variance in system function that is explained by a perturbation. This approach can provide evidence in support of a causal theory [151], but does not itself comprise a causal framework. In contrast, impulse response models and network control models apply an artificial perturbation in silico to predict the effects of a natural perturbation in vivo. Two causal frameworks that appear particularly relevant to these models are that of mechanisms and that of pathways [152, 153]. Future work bridging empirical science, theoretical science, and philosophy of science could seek to clarify particularly fruitful points of interdigitation between perturbative models and causal frameworks. Moreover, such efforts could delineate best practices in causal inference from observational studies with particularly abundant data versus from clinical studies with less data and carefully designed interventions.

Finally, it is worth posing the question of whether computational models are the end goal, or whether they are a humble stepping stone to a larger goal. In considering this question, we are faced with the fundamental differences and relations between modeling and theory. What we truly seek is understanding, and computational models (along with their associated analytical approaches) are useful tools with which to gain understanding. But a model is not the understanding itself. Models can instantiate theories, and understanding arises when theories are tested, and either proven or disproven. An important future direction, then, is to define the theories behind the models, and use the models in a more directed way to prove or disprove specific theories.

## Conclusion

Together, our goal is to understand how, when, and where to intervene to divert mental illness and drive mental health. Frankly, we are reaching for the stars. While the stars may still be beyond our reach, we may attain some altitude by taking a path through the Dancing Forest, and acknowledging how the wind bends the trees. Recent advances in modeling the relations between perturbations and system function lay the groundwork for us to better understand how brain, symptom, and behavior systems respond to exogenous changes both in health and disease.

## Citation diversity statement

Recent work in several fields of science has identified a bias in citation practices such that papers from women and other minorities are under-cited relative to the number of such papers in the field [154–159]. Here we sought to proactively consider choosing references that reflect the diversity of the field in thought, form of contribution, gender, and other factors. We obtained predicted gender of the first and last author of each reference by using databases that store the probability of a name being carried by a woman [154, 160]. By this measure (and excluding self-citations to the first and last authors of our current paper), our references contain 12% woman(first)/woman(last), 14.5% man/woman, 19.7% woman/man, 53.8% man/man, and 0% unknown categorization. This method is limited in that (a) names, pronouns, and social media profiles used to construct the databases may not, in every case, be indicative of gender identity and (b) it cannot account for intersex, non-binary, or transgender people. We look forward to future work that could help us to better understand how to support equitable practices in science.

## Funding and disclosure

D.M.L.-S. was supported by a K01 from the National Institute on Drug Abuse (K01DA047417). E.J.C. was supported by an F30 from the National Institute of Mental Health (F30 MH118871-01). A.S.B. and D.S.B. acknowledge support from the John D. and Catherine T. MacArthur Foundation, the ISI Foundation, the Paul G. Allen Family Foundation, the Army Research Laboratory (W911NF-10-2-0022), the Army Research Office (Falk W911NF-18-1-0244, Bassett-W911NF-14-1-0679, Grafton-W911NF-16-1-0474), the National Science Foundation (BCS1631550, PHY-1554488, NCS-FO-1926829), the National Institute of Mental Health (2-R01-DC-009209-11, R01-MH112847, R01-MH107235, R21-M MH-106799, R01-MH-116920), and the National Institute of Child Health and Human Development (1R01HD086888-01). The content is solely the responsibility of the authors and does not necessarily represent the official views of any of the funding agencies. The authors declare no competing interests.
